# Associations between *PPARG* polymorphisms and the risk of essential hypertension

**DOI:** 10.1371/journal.pone.0181644

**Published:** 2017-07-20

**Authors:** Gaojun Cai, Xinyong Zhang, Weijin Weng, Ganwei Shi, Sheliang Xue, Bifeng Zhang

**Affiliations:** 1 Department of Cardiology, Wujin hospital affiliated to Jiangsu University, Changzhou, Jiangsu Province, China; 2 Department of Neurology, Huai’an second people’s hospital, the affiliated Huai’an hospital of Xuzhou Medical University, Huaian, Jiangsu Province, China; 3 Department of Pathology and Molecular Medicine, McMaster University, Ontario, Canada; Children's National Health System, UNITED STATES

## Abstract

**Background:**

Peroxisome proliferator-activated receptor gamma (*PPARG*) plays an important role in the pathogenesis and maintenance of essential hypertension (EH). It has been suggested that polymorphisms of *PPARG* are associated with the risk of EH. However, findings to date remain controversial. To elucidate the associations between the *PPARG* Pro12Ala and C161T polymorphisms and EH risk, a meta-analysis was carried out.

**Methods:**

A comprehensive literature search of PubMed, Embase, CNKI (Chinese National Knowledge Infrastructure), VIP and Wanfang databases was conducted. The pooled odds ratios (ORs) and 95% confidence interval (CI) were calculated to estimate the size of the effect using the random-effects model. At the same time, the pooled standardized mean difference (SMD) with 95% CI was used for the meta-analysis of the *PPARG* Pro12Ala polymorphism and blood pressure.

**Results:**

Finally, Fifteen papers (seventeen studies) including 4,151 cases and 4,997 controls to evaluate the association of the *PPARG*Pro12Ala polymorphism and EH risk, were included in this study. Overall, the results suggested that Ala allele was associated with the decreased EH risk (for allelic model, OR = 0.757, 95%CI: 0.624–0.918, *P* = 0.005; for dominant model, OR = 0.771, 95%CI: 0.627–0.946, *P* = 0.013). The subgroup analysis stratified by ethnicity showed that the significant association between the *PPARG* Pro12Ala polymorphism and EH was only detected in the Asian subgroup. There was no difference in blood pressure values between Ala carriers and non-carriers. For the C161T polymorphism, only 5 studies comprising 1,118 cases and 1,357 controls met the inclusion criteria. The overall results showed that the *PPARG* C161T polymorphism was not associated with the risk of EH. But in the subgroup analysis, we found that the *PPARG* C161T polymorphism significantly associated with the risk of EH in the Asian subgroup (for allelic model, OR = 0.719, 95% CI: 0.537–0.963, *P* = 0.027; for dominant model, OR = 0.653, 95% CI: 0.439–0.972, *P* = 0.036).

**Conclusion:**

Our meta-analysis suggested that the *PPARG* polymorphisms might be associated with the risk of EH.

## Background

Essential hypertension (EH) is one of the leading causes of mortality and morbidity. Epidemiological data show that there are about one billion EH patients in the world. Pathogenesis of EH is related to multiple risk factors, including environmental and genetic factors [1, 2]. Systematic reviews and meta-analyses have independently suggested that numerous gene polymorphisms are associated with the risk of EH [2, 3]. Genetic factors might contribute 30–50% to the pathogenesis of EH [4].

Peroxisome proliferator-activated receptor gamma (*PPARG*, also known as *NR1C3*), a member of the nuclear hormone receptor subfamily, regulates the expression of a network of genes involved in adipogenesis and lipogenesis, insulin sensitivity, inflammation and atherosclerosis [5]. *PPARGs* consist of three different isoforms-*PPARG*1, *PPARG*2 and *PPARG*3-result different promoter and different splicing methods. *PPARG* is mainly expressed in adipocyte tissue, as well in vascular endothelial cells, smooth muscle cells and monocyte/macrophage cells [6]. Previous studies showed that *PPARG* plays an important role in adipose differentiation and in susceptibility to type 2 diabetes [7]. In recent years, decreased *PPARG* levels have been reported in subjects with high blood pressure not only in vitro but also in vivo [8].

The gene encoding *PPARG* is located in human chromosome 3p25 and contains 9 exons. Several *PPARG* polymorphisms have recently been identified. Studies have shown that *PPARG* polymorphisms might be associated with coronary artery disease [9], type 2 diabetes [10] and metabolic syndrome [11].

To date, numerous studies have been conducted to explore the relationship between *PPARG* polymorphisms and the risk of EH in different populations, but the results have been conflicting. One of the common variants in *PPARG* is Pro12Ala (*rs1805192*). The cytosine is substituted for guanosine at nucleotide position 34 (C34G), leading to a change from proline (Pro) to alanine (Ala) at position 12 of exon 2 in *PPARG*. This substitution may lead to the change of *PPARG* protein structure, which can decrease the effect of binding to the target gene and reduce its transcriptional activity [12]. In previous studies, the Ala allelic frequency varied greatly in different ethnic populations [13, 14], which could be attributed to genetic variations and to different environmental and lifestyle exposures.

Studies on the association of *PPARG* Pro12Ala polymorphism with EH risk have been extensively performed previously, but the results remain controversial [15, 16, 17]. In 2008, Lu et al [18] explored the relationship between the Pro12Ala variant and EH among long-lived subjects (more than 90 years). The mean age of included subjects was 94 years. They found that the frequency of the Ala allele was significantly lower in the EH group than in the normotensive group (3.45% *vs*. 6.92%, *P* = 0.001). Several other studies reached similar conclusions [15, 19]. However, Horiki et al [20] did not find an association between the *PPARG*Pro12Ala polymorphism and EH. More contrary results were found in other studies [21–24]. In 2006, Stefanski et al [21] examined the association between the Pro12Ala polymorphism and blood pressure values in obese patients with long-lasting type 2diabetes in Poland. They found that carriers with the Ala allele had a higher 24h diastolic pressure than patients with Pro/Pro genotype. In 2010, Gao et al [23] concluded that the Ala allele was involved in genetic susceptibility to EH in the Han population of Inner Mongolia.

Another variant is C161T (*rs3856806*), which is located in the exon6 of *PPARG*. As for association between the C161T polymorphism and the risk of EH, the results are also inconsistent. For example, Qu et al [24] found that the frequency of the CT genotype was significantly lower in EH patients than in normotensive subjects (21.3% *vs*. 36.2%, *P*<0.05) and that the T allele protected one from EH. In 2014, Zhang et al [25] obtained a similar result. However, other studies concluded that the *PPARG* C161T polymorphism was not associated with EH susceptibility [24, 26].

To clarify these inconsistent findings and to evaluate the contribution of *PPARG* polymorphisms to the risk of EH, we performed a meta-analysis based on available data.

## Material and methods

### Study selection

The meta-analysis followed the Preferred Reporting Items for Systematic Reviews and Meta-analysis criteria [27]. A comprehensive literature search of PubMed, Embase, CNKI (Chinese National Knowledge Infrastructure), VIP and Wanfang databases was conducted by two investigators independently before November 2016. The following search terms were used in the electronic searches: (“PPAR” or “peroxisome proliferator-activated receptor” or “*NR1C3*”) and (“polymorphism” or “variant” or “gene” or “mutation”) and (“essential hypertension” or “high blood pressure” or “EH”). For example, the full search strategy used in the PubMed database is: text word = (“PPAR” or “peroxisome proliferator-activated receptor” or “*NR1C3*”) and (“polymorphism” or “variant” or “gene” or “mutation”) and (“essential hypertension” or “hypertension” or “high blood pressure” or “EH”). To find additional eligible studies, the reference lists of the studies included were searched manually.

### Inclusion and exclusion criteria

Eligible studies had to meet the following inclusion criteria: 1) Studies on the association between *PPARG* polymorphisms and EH risk; 2) Case-control study; 3) Having clear, original data of genotypic and/or allelic frequencies; 4) Studies written in English and Chinese; 5) Hypertension being defined as systolic blood pressure ≥140 mmHg and/or diastolic blood pressure ≥90 mmHg or treatment with anti-hypertensive medication; 6) For blood pressure, the data being presented as the mean± standard deviations (SD).

The exclusion criteria were as follows: 1) a review or case report; 2) genotypic and allelic frequencies were not clear; 3) animal studies; 4) secondary hypertension; 5) cross-sectional or cohort study; 6) data published repeatedly.

### Data extraction

A special table was used to record the following information: the name of first author, year of publication, country, ethnicity, age, type of study, source of control, genotyping methods, the distribution of genotypes in cases and controls, diagnostic criteria of EH, and the value of blood pressures. The original data were extracted by two investigators (Cai GJ and Zhang BF) independently. Disagreements were resolved by consulting with a third author (Weng WJ). If we were in doubt about the data in the study, we tried our best to contact the author for correspondence by email.

### Statistical analysis

We used Stata 12.0 (StataCorp LP, College Station, Texas, USA) software for all the statistical analysis. Hardy-Weinberg equilibrium (HWE) for the *PPARG* genotype distributions of control groups was checked by Fisher’s exact test. Pooled odds ratios (ORs) and 95% confidence interval (CI) were used to assess the strength of association between *PPARG* polymorphisms and EH risk, and the pooled standardized mean difference (SMD) with 95% CI was used for the meta-analysis of *PPARG* polymorphisms and blood pressure. The between-study heterogeneity was evaluated by the Chi-square-based Cochrane’s Q statistic and the *I*^2^ statistic. If the between-study heterogeneity was significant (*P*-value< 0.10 or *I*^2^> 50%), a random effect model (Dersimonian-Laird method) was used to calculate the result; otherwise, a fixed effect model (Mantel-Haenszel method) was used [28]. Due to the small number of minor alleles, only the dominant (for Pro12Ala, GG+GC *vs*. CC; for C161T, TT+TC *vs*. CC) and allelic (for Pro12Ala, G *vs*. C; for C161T, T *vs*. C) model were performed to calculate the pooled ORs in the present meta-analysis. Subgroup analyses were also conducted by ethnicity (Caucasian and Asian), according to HWE (Yes and No), source of control (hospital based and population based) and genotyping methods (PCR-RFLP and others) to explore the source of heterogeneity. A sensitivity analysis omitting an individual study each time was used to evaluate the stability of the main meta-analysis results. The potential publication bias was examined by Begg’s funnel plot, and the funnel plot asymmetry was assessed by Egger’s linear regression test. A*P-*value <0.05 (two-sided) was considered statistically significant.

## Results

### Study characteristics

**Fig 1:**
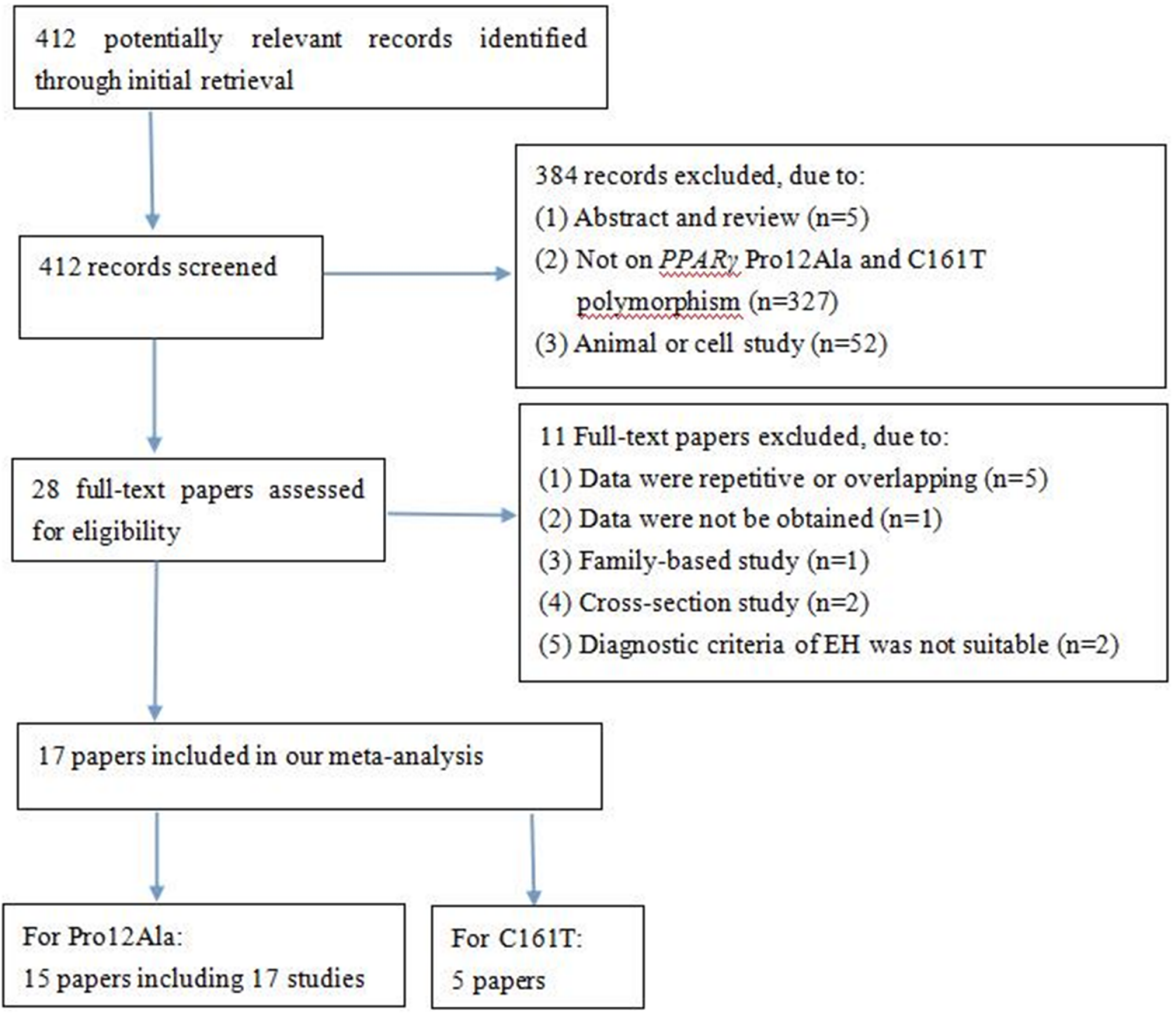
Flow diagram of paper selection.

A total of 412 potentially relevant papers were obtained by the literature search, of which 17 papers met the inclusion criteria. **Fig 1** lists the flow diagram of the article selection process. The 17 studies (15 papers) evaluated the *PPARG* Pro12Ala polymorphism and EH risk, including 4,151 cases and 4,997 controls [13–16, 18–20, 23, 26, 29–34]. Among these papers, two articles [31, 33] contained two subgroups with two independent results, respectively, so we analysed each as two separate studies. Most of the studies were conducted in Asians, with only three studies in Caucasians. The Ala allelic frequency in controls ranged from 0.28% in the Dong HR et al. study to 29.0% in the study by Wang et al. Five studies evaluated the *PPARG* C161T polymorphism and EH risk, including 1,118 cases and 1,357 controls [23–26, 34]. All studies were conducted in Chinese, except for the Grygiel-Górniak et al. study. **Tables 1 and 2** summarise the characteristics of included articles, including ethnicity, year of publication, genotyping method, distribution of genotypes and alleles, etc. The diagnostic criteria of EH were appropriated in all of these studies. The controls in 3 studies deviated from HWE [31, 32]. Six papers evaluated the relationship between the *PPARG* Pro12Ala polymorphism and the value of systolic blood pressure (SBP) and diastolic blood pressure (DBP) (**Table 3**). Of those, five papers were conducted in Asians and one paper in Caucasians. Because only two papers studied the relationship between the *PPARG*C161T polymorphism and blood pressure values, we did not perform meta-analysis.

**Table 1:** Main characteristics of studies involved in this meta-analysis of the *PPARG* Pro12Ala polymorphism and essential hypertension risk.

First author	Year	Country	Ethnicity	Source of control	Mean age (y) (case/control)	Sample size (case/control)	Cases			Controls			Genotyping methods	MAF (%)	HWE (Y/N)
							Pro/Pro	Pro/Ala	Ala/Ala	Pro/Pro	Pro/Ala	Ala/Ala			
Rodríguez-Esparragón FJ	2003	Spain	Caucasian	HB	59±9/ 57±8	229/ 212	206	22	1	174	36	2	PCR-RFLP	9.43	Y
Horiki M	2004	Japan	Asian	HB	-/ -	205/ 300	193	12	0	276	24	0	PCR-RFLP	4.00	Y
Shen D	2005	China	Asian	HB	69.2±13.5/ 67.3±11.7	70/ 220	66	3	1	206	13	1	PCR-RFLP	3.41	Y
Zhang AP	2005	China	Asian	HB	58.8±10.7/ 56.7±11.2	132/ 157	128	4	0	148	9	0	PCR-RFLP	2.87	Y
Gouni-Berthold I	2005	Germany	Caucasian	HB	64.6+9.9/ 60.3±11.8	255/ 140	190	57	8	104	32	4	PCR-RFLP	14.29	Y
Pan HJ	2007	China	Asian	PB	50.83±12.10/ 39.79±9.67	177/ 119	154	23	0	101	18	0	PCR-RFLP	7.56	Y
Yin RX ^*^	2008	China	Asian	PB	51.2±14.2/ 43.4±15.2	287/ 547	265	17	5	518	24	5	PCR-RFLP	3.11	N
Yin RX^Δ^	2008	China	Asian	PB	55.2±12.1/ 43.8±15.2	159/ 676	153	6	0	629	40	7	PCR-RFLP	3.99	N
Lu ZC	2008	China	Asian	PB	94.5±4.0/ 94.8±4.0	478/ 361	446	31	1	312	48	1	PCR-RFLP	6.93	Y
Gao L	2010	China	Asian	HB	54.47±16.21/50.08±15.01	345/ 137	337	7	1	131	2	4	PCR-RFLP	3.65	N
Dong HR	2012	China	Asian	HB	48.60±13.33/ 38.48±10.93	124/ 178	122	2	0	177	1	0	PCR-RFLP	0.28	Y
Lin Y	2012	China	Asian	PB	52.10±9.87/ 49.04±9.02	269/ 551	166	90	16	293	205	50	TaqMan	28.22	Y
Zhang XF^δ^	2013	China	Asian	HB	51.68±9.63/ 51.87±11.18	146/ 112	125	20	1	101	11	0	PCR-RFLP	4.91	Y
Zhang XF ^&^	2013	China	Asian	HB	51.76±8.36/ 50.31±8.18	163/ 178	137	25	1	18	20	0	PCR-RFLP	5.61	Y
Chen J	2014	China	Asian	PB	45.05±12.86/ 42.37±11.61	144/ 165	110	33	2	105	53	7	MALDI-TOF-MS	20.3	Y
Wang G	2015	China	Asian	PB	52.8±16.2/ 51.4±17.6	816/ 824	536	244	36	426	318	80	PCR-RFLP	29.00	Y
Grygiel-Górniak B	2015	Poland	Caucasian	HB	59.88±5.07/58.59±5.86	151/ 120	101	44	6	84	32	4	TaqMan	16.67	Y

Abbreviations: MAF, minor allelic frequency; HWE, Hardy-Weinberg equilibrium; HB, hospital based; PH, population based; PCR-RFLP, polymerase chain reaction-restriction fragment length polymorphism

*male subgroup

Δ female subgroup

δ, Hui subgroup

&, Chinese Han subgroup

Y, yes; N, no.

**Table 2:** Main characteristics of studies involved in this meta-analysis of the *PPARG* C161T polymorphism and essential hypertension risk.

First author	Year	Country	Ethnicity	Source of control	Mean age (y) (case/control)	Sample size (case/control)	Cases			Controls			Genotyping methods	MAF (%)	HWE (Y/N)
							CC	CT	TT	CC	CT	TT			
Qu FZ	2005	China	Asian	HB	49.0±10.0/ 46.0±12.0	160/ 116	121	34	5	70	42	4	PCR-RFLP	21.55	Y
Lin Y	2012	China	Asian	PB	52.1±9.9/ 49.0±9.0	269/ 551	140	108	21	278	218	55	TaqMan	29.76	Y
Chen J	2014	China	Asian	PB	45.1±12.9/ 42.4±11.6	144/ 165	90	49	5	96	64	5	MALDI-TOF-MS	22.42	Y
Zhang Y	2014	China	Asian	HB	63.9±10.8/ 63.3±9.5	393/ 405	270	108	15	204	177	24	PCR-RFLP	27.78	Y
Grygiel-Górniak B	2015	Poland	Caucasian	HB	59.9±5.1/ 58.6±5.9	151/ 120	107	39	5	91	27	2	PCR-RFLP	12.92	Y

MAF, minor allelic frequency; HWE, Hardy-Weinberg equilibrium; PCR-RFLP, polymerase chain reaction-restriction fragment length polymorphism;HB, hospital based; PH, population based; Y, yes; N, no.

**Table 3:** Characteristics of individual studies included in the meta-analysis of the *PPARG* Pro12Ala polymorphism and blood pressure.

First author	Year	Country	Ethnicity	Subpopulation	Genotypes	Number (n)	SBP		DBP	
							M	SD	M	SD
Zhang AP	2005	China	Asian	All	Pro/Pro	276	158.6	25.1	95.6	15.3
					Pro/Ala	13	132.3	16.7	91.3	12.5
Shen D	2005	China	Asian	EH	Pro/Pro	66	155.6	13.5	90.3	7.2
					Pro/Ala+Ala/Ala	4	159.3	15.8	91.7	7.6
Gao L	2010	China	Asian	EH	Pro/Pro	337	147.8	15.4	88.1	11.2
					Pro/Ala+Ala/Ala	8	150.8	21.3	88.3	11.1
				Control	Pro/Pro	131	111.9	11.0	73.3	9.1
					Pro/Ala+Ala/Ala	6	120.5	14.5	71.7	10.1
Zhang XF ^δ^	2013	China	Asian	EH	Pro/Pro	125	155.6	11.6	92.1	11.9
					Pro/Ala+Ala/Ala	21	154.7	10.3	91.8	9.1
				Control	Pro/Pro	101	117.5	10.2	74.9	8.4
					Pro/Ala	11	115.0	15.9	74.6	9.3
Zhang XF ^&^	2013	China	Asian	EH	Pro/Pro	137	153.7	15.4	94.1	9.8
					Pro/Ala+Ala/Ala	26	155.4	17.49	95.2	9.6
				Control	Pro/Pro	158	123.7	10.2	75.4	7.5
					Pro/Ala	20	126.4	7.0	76.7	7.1
Grygiel-Górniak B	2015	Poland	Caucasian	EH	Pro/Pro	101	158.2	15.0	96.8	11.4
					Pro/Ala	44	155.3	15.6	94.8	12.1
					Ala/Ala	6	158.7	20.6	104.8	10.0
				Control	Pro/Pro	84	122.3	10.0	77.8	6.9
					Pro/Ala	32	119.0	12.0	76.1	6.7
					Ala/Ala	4	117.5	10.7	79.0	5.6

δ, Hui subgroup

&, Chinese Han subgroup

EH, essential hypertension; SBP, systolic blood pressure; DBP, diastolic blood pressure; M, mean; SD, standard deviations

### Meta-analysis results

#### Association of *PPARG* Pro12Ala polymorphism with EH risk

**Fig 2:**
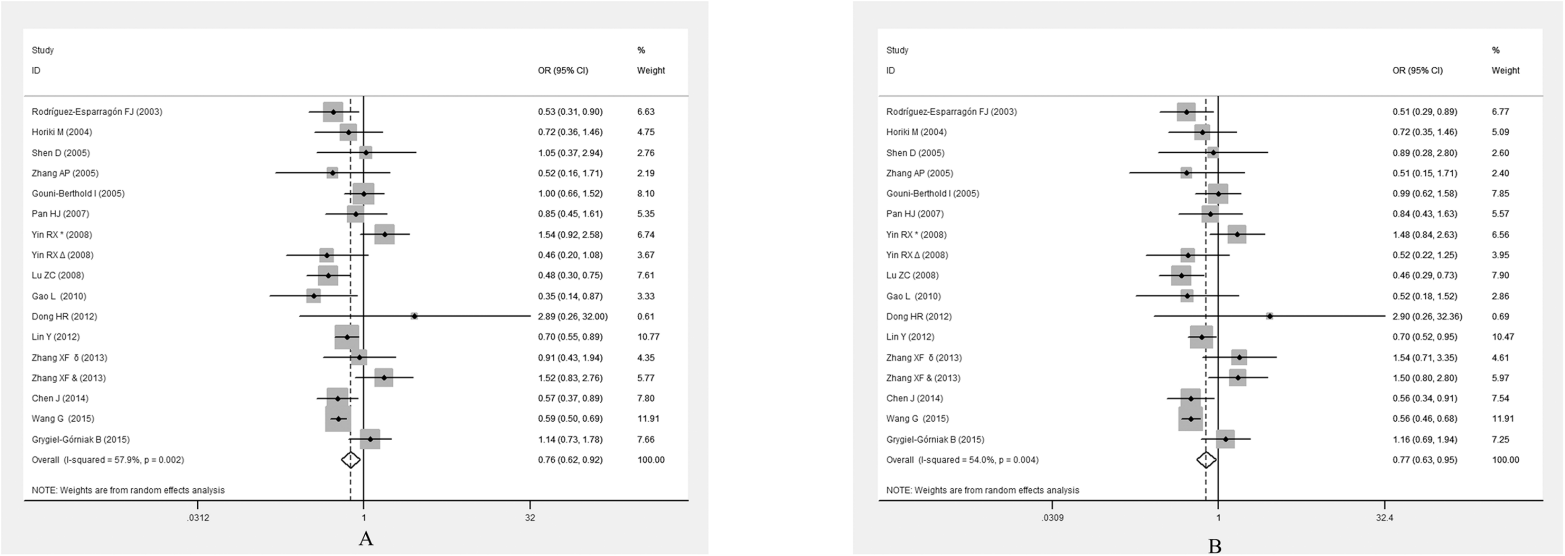
Forest plot for the *PPARG* Pro12Ala polymorphism and EH risk under the allelic model (A)and dominant model (B).

**Table 4** summarises the association between the *PPARG*Pro12Ala polymorphism and the risk of EH. Because between-study heterogeneity was significant in the overall analysis, a random-effect model was used. There were significant associations between the *PPARG* Pro12Ala polymorphism and EH risk in the whole population under allelic (OR = 0.757, 95% CI: 0.624–0.918,*P* = 0.005) and dominant genetic models (OR = 0.771, 95% CI: 0.627–0.946, *P* = 0.013) (**Fig 2**).

**Table 4:** Summary of meta-analysis of association of the *PPARG* polymorphisms and essential hypertension risk.

SNPs	Subgroup	Dominant model					Allelic model			
		N	OR (95% CI)	*P*_OR_	*I*^2^ (%)	*P*_Q_	OR (95% CI)	*P*_OR_	*I*^2^ (%)	*P*_Q_
**Pro12Ala**	All	17	0.771 (0.627–0.946)	**0.013**	54.0	0.004	0.757 (0.624–0.918)	**0.005**	57.9	0.002
	Ethnicity									
	Asian	14	0.749 (0.595–0.944)	**0.014**	52.5	0.011	0.726 (0.587–0.887)	**0.003**	54.6	0.007
	Caucasian	3	0.847 (0.531–1.351)	0.486	59.6	0.084	0.865 (0.563–1.329)	0.509	61.6	0.074
	Genotyping method									
	PCR-RFLP	13	0.826 (0.623–1.095)	0.184	48.0	0.027	0.790 (0.596–1.047)	0.101	53.9	0.011
	Others	4	0.676 (0.515–0.889)	**0.005**	59.7	0.059	0.690 (0.543–0.879)	**0.003**	63.2	0.043
	Source of control									
	HB	10	0.921 (0.697–1.217)	0.562	28.1	0.186	0.863 (0.652–1.141)	0.301	37.7	0.108
	PB	7	0.658 (0.514–0.946)	**0.001**	55.1	0.038	0.675 (0.532–0.855)	**0.001**	62.3	0.014
	HWE									
	*P*>0.05	14	0.752 (0.609–0.928)	**0.008**	52.3	0.012	0.745 (0.620–0.894)	**0.002**	49.4	0.019
	*P*<0.05	3	0.805 (0.370–1.750)	0.584	62.6	0.009	0.664 (0.244–1.809)	0.423	81.3	0.005
**C161T**	All	5	0.734 (0.499–1.081)	0.117	78.9	0.001	0.791 (0.587–1.064)	0.121	75.0	0.003
	Ethnicity									
	Asian	4	0.653 (0.439–0.972)	**0.036**	78.1	0.003	0.719 (0.537–0.963)	**0.027**	72.2	0.013
	Caucasian	1	1.290 (0.748–2.227)	0.36	0	0	1.306 (0.807–2.122)	0.282	0	0
	Genotyping method									
	PCR-RFLP	3	0.646 (0.354–1.178)	0.154	81.7	0.004	0.727 (0.444–1.191)	0.206	79.7	0.007
	Others	2	0.907 (0.709–1.160)	0.438	0	0.673	0.907 (0.745–1.104)	0.329	0	0.919
	Source of control									
	HB	3	0.646 (0.354–1.178)	0.154	81.7	0.004	0.727 (0.444–1.191)	0.206	79.7	0.007
	PB	2	0.907 (0.709–1.160)	0.438	0	0.673	0.907 (0.745–1.104)	0.329	0	0.919

N, number of study; HWE, Hardy-Weinberg equilibrium; PCR-RFLP, polymerase chain reaction-restriction fragment length polymorphism;HB, hospital based; PH, population based.

**Fig 3:**
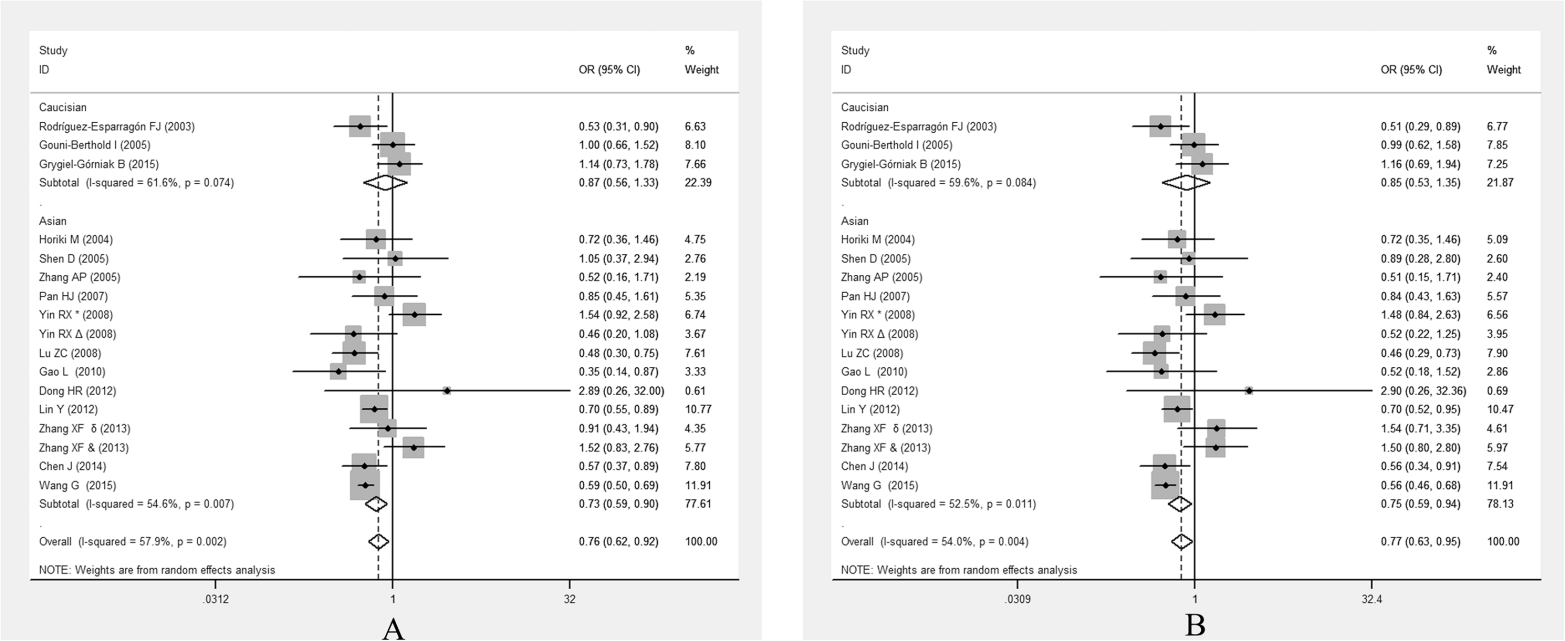
Forest plots of ORs for the association between the *PPARG* Pro12Ala polymorphism and susceptibility to EH in subgroup analysis based on ethnicity under the allelic model (A) and the dominant model (B).

In the subgroup analysis stratified by ethnicity, the significant association between *PPARG* Pro12Ala polymorphism and EH was only detected in Asian population under allelic (OR = 0.726, 95% CI: 0.587–0.887, *P* = 0.003) and dominant genetic models (OR = 0.749, 95% CI: 0.595–0.944, *P* = 0.014) (**Fig 3**).

**Fig 4:**
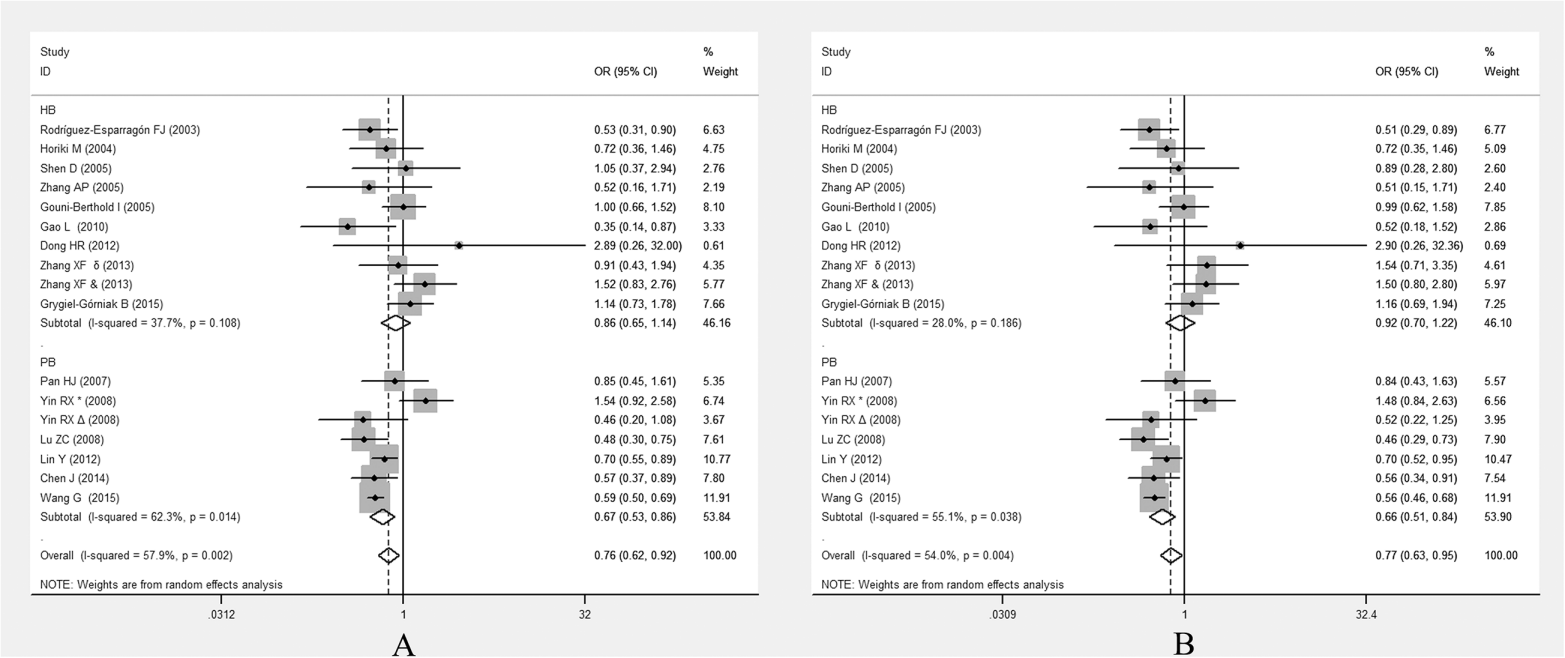
Forest plots of ORs for the association between the *PPARG*Pro12Ala polymorphism and susceptibility to EH in subgroup analysis based on source of control under the allelic model (A) and the dominant model (B).

*PPARG* Pro12Ala polymorphism was associated with EH in a population-based subgroup (for allelic model, OR = 0.675, 95% CI: 0.532–0.855, *P* = 0.001; for dominant model, OR = 0.658, 95% CI: 0.514–0.946, *P* = 0.001)(**Fig 4)**.

In the subgroup, based on whether the control genotype distribution displayed HWE, a significant association was found between the *PPARG* Pro12Ala polymorphism and EH under allelic (OR = 0.745, 95% CI: 0.620–0.894, *P* = 0.002) and dominant genetic models (OR = 0.752, 95% CI: 0.609–0.928, *P* = 0.008) (**S1 Fig**).

Stratification based on the genotyping method revealed no significant association between the *PPARG* Pro12Ala polymorphism and EH risk in the PCR-RFLP subgroup, whereas a statistically significant association was found in the other methods subgroup (for allelic model, OR = 0.690, 95% CI: 0.543–0.879, *P* = 0.003; for dominant model, OR = 0.676, 95% CI: 0.515–0.889, *P* = 0.005) (**S2 Fig**).

As shown in **S3 Fig**, there was no significant difference in the value of blood pressure between Ala carriers and non-carriers (for SBP, SMD: 0.08, 95% CI: -0.18–0.33, *P* = 0.562; for DBP, SMD: 0.04,95% CI: -0.11–0.20, *P* = 0.594, respectively).

#### Association of *PPARG* C161T polymorphism with EH risk

**Fig 5:**
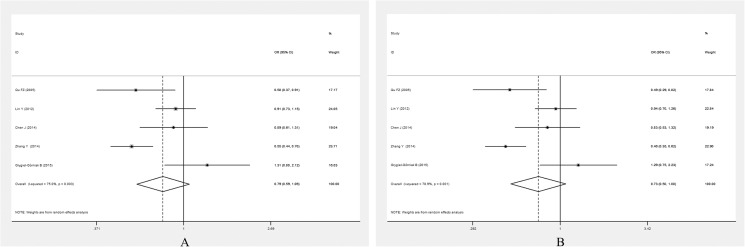
Forest plot of the *PPARG*C161T polymorphism and EH risk under the allelic model (A) and dominant model (B).

As shown in **Fig 5**, there was no significant association between the *PPARG* C161T polymorphism and EH risk in the whole population under allelic (OR = 0.791, 95% CI: 0.587–1.064, *P* = 0.121) and dominant genetic models (OR = 0.734, 95% CI: 0.499–1.081, *P* = 0.117).

**Fig 6:**
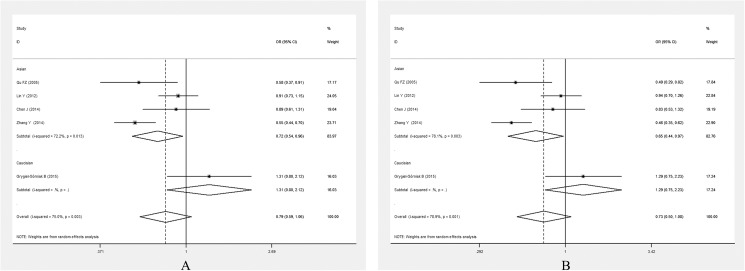
Forest plots of ORs for the association between the *PPARG*C161T polymorphism and susceptibility to EH in subgroup analysis based on ethnicity under the allelic model (A) and the dominant model (B).

Because the between-study heterogeneity was also significant in the whole study (for allelic model, *I*^2^ = 75.0%, *P* = 0.003; for dominant model, *I*^2^ = 78.9%, *P* = 0.001), so subgroup analyses stratified by ethnicity, genotyping methods and source of control were also conducted. The subgroup analyses showed that the statistically significant association only existed in the Asian subgroup (for allelic model, OR = 0.719, 95% CI: 0.537–0.963, *P* = 0.027; for dominant model, OR = 0.653, 95% CI: 0.439–0.972, *P* = 0.036) (**Fig 6**).

### Sensitivity analysis

**Fig 7:**
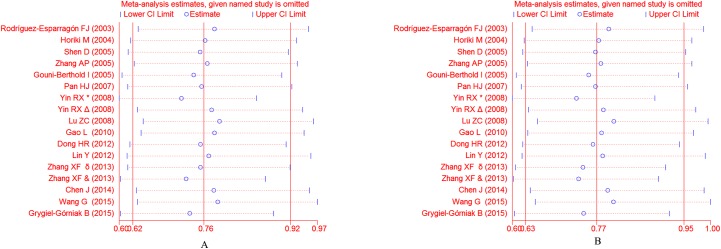
Sensitivity analyses of the association between the *PPARG* Pro12Ala polymorphism and susceptibility to EH under the allelic model (A) and the dominant model (B).

**Fig 8:**
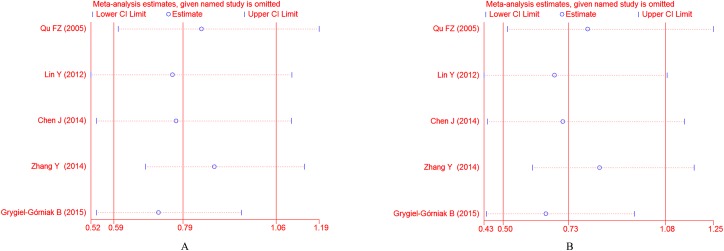
Sensitivity analyses of the association between the *PPARG*C161T polymorphism and susceptibility to EH under the allelic model (A) and the dominant model (B).

Because of the significant between-study heterogeneity, sensitivity analysis was conducted to determine the influence of individual studies on pooled ORs by sequentially removing each eligible study. No single study of the Pro12Ala polymorphism influenced the stability of the entire study (Fig 7). For the C161T polymorphism, The study by Grygiel-Górniak et al. significantly affected the overall results (**Fig 8**). When the Grygiel-Górniak et al. study was excluded, the pooled result changed to the contrary.

### Publication bias

**Fig 9:**
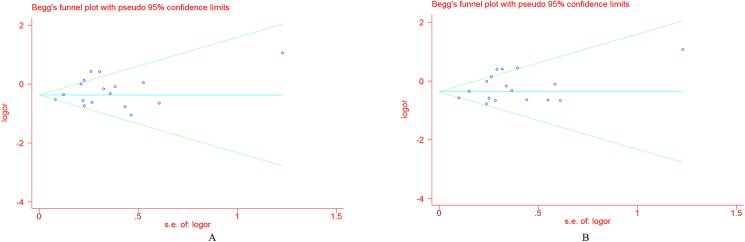
Funnel plot for the *PPARG* Pro12Ala polymorphism and EH risk under the allelic model (A) and the dominant model (B).

**Fig 10:**
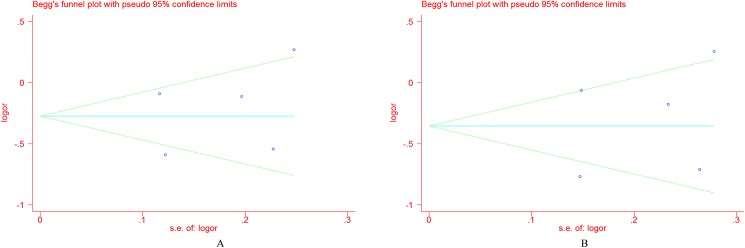
Funnel plot for the *PPARG*C161T polymorphism and EH risk under the allelic model (A) and the dominant model (B).

The Begg’s funnel plot and Egger’s test were adopted to evaluate the publication bias among the individual studies. For Pro12Ala, **Fig 9** showed that a visual publication bias was found in the Begg’s funnel plot under both allelic and dominant models. The significant difference was also detected by Egger’s test (*P* = 0.002 under the dominant model; *P* = 0.002 under the allelic model). For the C161T polymorphism, no visual publication bias was found in the Begg’s funnel plot under both of the genetic models(**Fig 10**), which was confirmed by the Egger’s test (dominant model, *P* = 0.414; allelic model, *P* = 0.376). No obvious publication bias was found in the Begg’s funnel plot for blood pressure (**S4 Fig**), which was also confirmed by Egger’s test (for SBP, *P* = 0.362; for DBP, *P* = 0.628).

## Discussion

In the present meta-analysis, we evaluated the relationship between *PPARG* polymorphisms and the risk of EH. Our meta-analysis found associations of the Pro12Ala and C161T polymorphisms of *PPARG* with significant risk of EH.

Studies suggest that *PPARG* modulates the expression of genes involved in energy storage and utilisation [35] and plays a critical role in the regulation of adipocyte differentiation, lipogenesis, insulin resistance, inflammatory response, angiogenesis and atherosclerosis.

Clinical and experimental studies have indicated that *PPARG* might be associated with EH risk and the activation of central°PPAR-γ could attenuate angiotensin II-induced°hypertension [36]. The mechanism by which *PPARG* decreases blood pressure is still not completely clarified. The likely mechanisms are: 1) antagonism of the renin-angiotensin system, 2) inhibition vascular cell proliferation; and 3) improvement endothelial function [37].

Up to now, several polymorphisms in *PPARG* had been studied, including Pro12Ala (*rs1805192*), C161T (*rs3856806*), C681G (*rs10865710*). As one of the important candidate genes for EH, studies on the association of *PPARG* polymorphisms and EH risk have been explored extensively. The results showed that *PPARG* polymorphisms might be associated with metabolism-related diseases [10]. In the present study, we selected the variant site in *PPARG* when it was in accord with the included criteria and was studied more than 3 times. After a literature search, the Pro12Ala and C161T polymorphisms in *PPARG* matched these conditions.

Although much research on the relationship between *PPARG*Pro12Ala and C161T polymorphisms and EH have been conducted so far, the results of individual studies were inconsistent. In 2003, a community-based, cross-sectional observational study to explore the relationship between the *PPARG* Pro12Ala polymorphism and blood pressure in male subjects with type 2 diabetes was performed in Swedish subjects. Subjects with the Pro/Ala or Ala/Ala genotype had significantly lower diastolic blood pressure. On the contrary, in a cross-sectional study conducted in the aboriginal Qatari population in 2013, Beneret al. [17] showed that participants with the Ala allele had a higher EH risk than those with the Pro allele. For the C161T polymorphism, this discrepancy also existed. The results of these studies were not consistent, which could be partly attributed to the insufficient power of the studies, different EH diagnostic criteria, and/or ethnic and environmental variations. For example, in the Kim et al. study, the authors defined EH as the systolic pressure greater than 130 mmHg and/ or diastolic pressure greater than 80 mmHg. In different countries, the discrepancy was remarkable. Even in the same countries, differences also existed among different ethnic groups. In China, this association was observed in Han [19] and Uyghur populations [23], rather than in Hui [33] and Zhuang populations [31].

In the present study, the results demonstrated that the *PPARG* Pro12Ala polymorphism was associated with susceptibility to EH under both allelic and dominant models, which was consistent with the study by Wang et al [38]. Although there was significant between-study heterogeneity, sensitivity analysis indicated that no single study influenced the pooled OR for the *PPARG*Pro12Ala polymorphism. Subgroup analysis stratified by ethnicity indicated that the association existed in Asians, but not Caucasians. However, there was no significant difference in the value of blood pressure between Ala carriers and the non-carriers. There was no significant association between the *PPARG* C161T polymorphism and EH risk in the whole population.However, in the subgroup analysis, we found that the *PPARG*C161T polymorphism was significantly associated with the risk of EH in the Asian subgroup. Sensitivity analysis showed that the Grygiel-Górniak et al. study significantly affected the overall results. When this study was excluded, the pooled result changed to the contrary. When we analysed this paper carefully, we found that this study was conducted in Caucasians and that the sample size was the smallest among the 5 papers, which contributed to the false-negative result.

In 2012, Wang et al. [38] conducted a meta-analysis to evaluate the relationship between the Pro12Ala polymorphism (*rs1801282*) in *PPARG* and EH susceptibility. They concluded that the Ala allele might be protective for hypertension among East Asians, but not among Caucasians. Compared with the study by Wang et al, the present meta-analysis had several strengths. First, not only Pro12Ala, but also C161T polymorphisms in *PPARG* were involved in this study, and the analysis of correlations between polymorphisms and EH were more comprehensively studied. Second, only 8 papers with 3,281 participants were involved in the study by Wang et al. in 2012. Since then, several new case-control studies on the relationship between *PPARG*Pro12Ala polymorphism and the susceptibility to EH were conducted. In the present meta-analysis, a total of 15 articles comprising 4,151 cases and 4,997 controls were involved, which increased the statistical power greatly. Third, due to the significant between-study heterogeneity, we performed sensitivity analysis and subgroup analysis to explore the source of heterogeneity.

Several limitations are present in our meta-analysis. First, as mentioned above, the association between Pro12Ala polymorphism and EH might be gender-specific. Because we could not obtain individual data from each study, we could not analyse the interaction between gene polymorphisms and the environment. Second, in our meta-analysis, most of the studies were conducted in Asians. There were only three studies conducted in Caucasians. Since the sample size for Caucasians was particularly small, the power was low, and thus results should be interpreted with caution. Third, only five studies with a small sample size (1,118 cases and 1,357 controls) were involved in our analysis of the association between the *PPARG* C161T polymorphism and susceptibility to EH. The sample size was relatively small, and any conclusion should be made cautiously. Finally, the publication bias was significant for the Pro12Ala polymorphism. A likely reason for the publication bias was that studies in other languages were not retrieved, and/or the negative studies were less likely to be published.

## Conclusion

In conclusion, our meta-analysis suggested that the *PPARG*Pro12Ala and C161T polymorphisms might be associated with the risk of EH in Asians. Since the Caucasian sample size was too small and statistically lacking in power, we should interpret it cautiously. Further large-scale studies should be conducted to confirm the above conclusions.

## Supporting information

S1 File PRISMA checklist. (DOC)

S2 File Meta-analysis-on-genetic-association-studies-form. (DOCX)

S1 Fig Forest plots of ORs for the association between the *PPARG* Pro12Ala polymorphism and susceptibility to EH in subgroup analysis based on HWE under the allelic model (A) and the dominant model (B).
(TIF)

S2 Fig Forest plots of ORs for the association between the *PPARG* Pro12Ala polymorphism and susceptibility to EH in subgroup analysis based on genotyping methods under the allelic model (A) and the dominant model (B).
(TIF)

S3 Fig Forest plot of the association between the *PPARG* Pro12Ala polymorphism and the value of systolic blood pressure (A) and diastolic blood pressure (B).
(TIF)

S4 Fig Funnel plot for the *PPARG* C161T polymorphism and the value of systolic blood pressure (A) and diastolic blood pressure (B).
(TIF)
