# Supplementary figures and images for: Associations between PPARG polymorphisms and the risk of essential hypertension
Source: PLoS One. 2017 Jul 20;12(7):e0181644. doi: 10.1371/journal.pone.0181644 (PMC5519177; doi:10.1371/journal.pone.0181644)

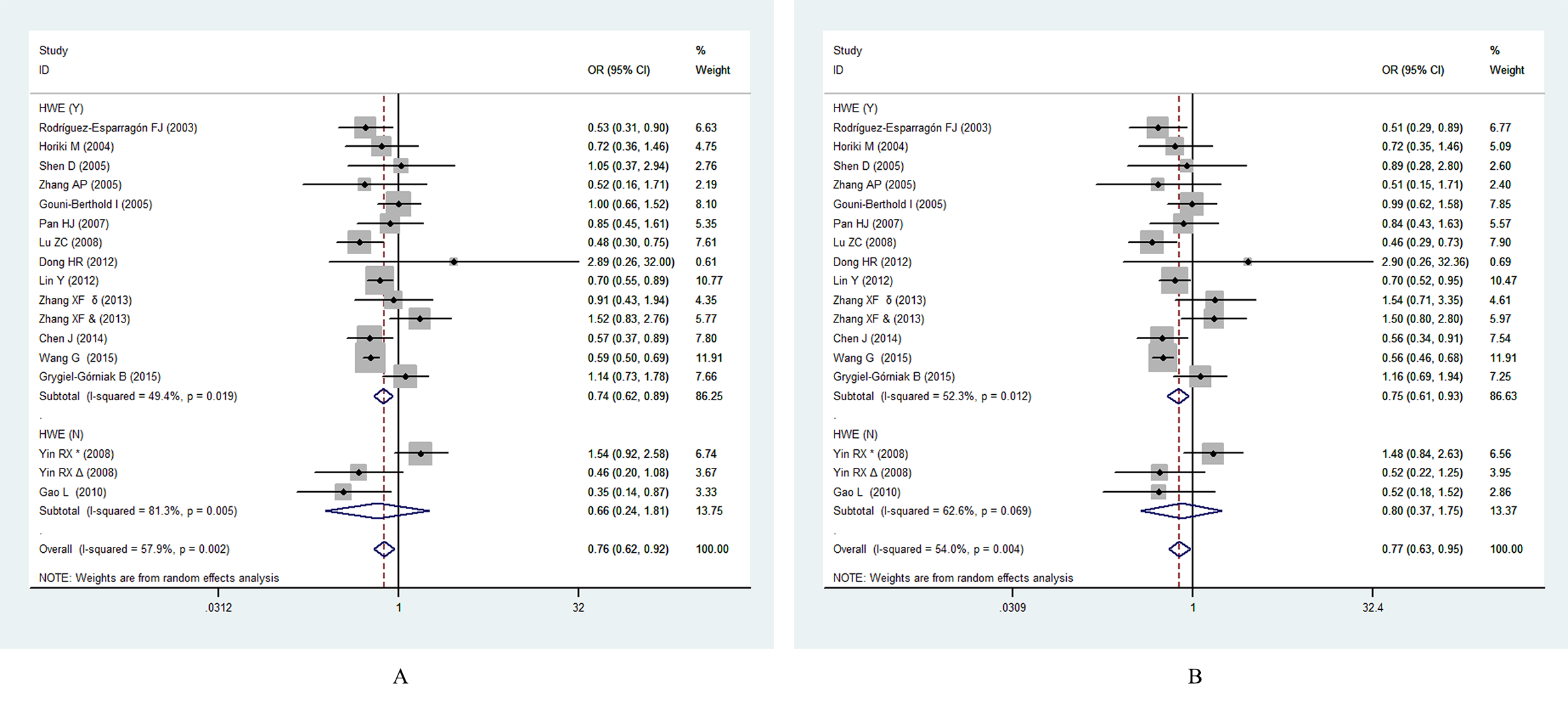

Supplement: S1 Fig — Forest plots of ORs for the association between the PPARG Pro12Ala polymorphism and susceptibility to EH in subgroup analysis based on HWE under the allelic model (A) and the dominant model (B). (TIF) [file pone.0181644.s003.tif]

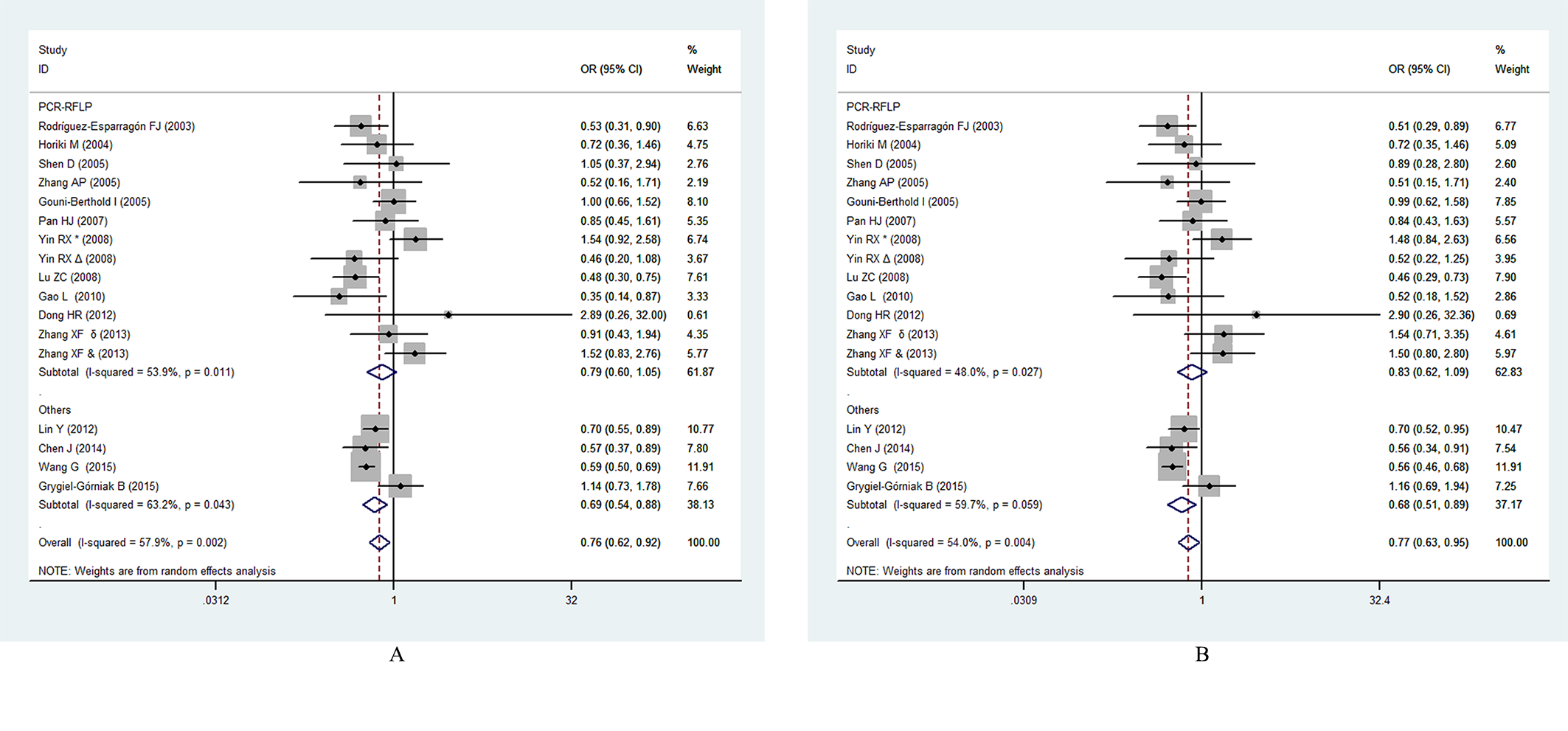

Supplement: S2 Fig — Forest plots of ORs for the association between the PPARG Pro12Ala polymorphism and susceptibility to EH in subgroup analysis based on genotyping methods under the allelic model (A) and the dominant model (B). (TIF) [file pone.0181644.s004.tif]

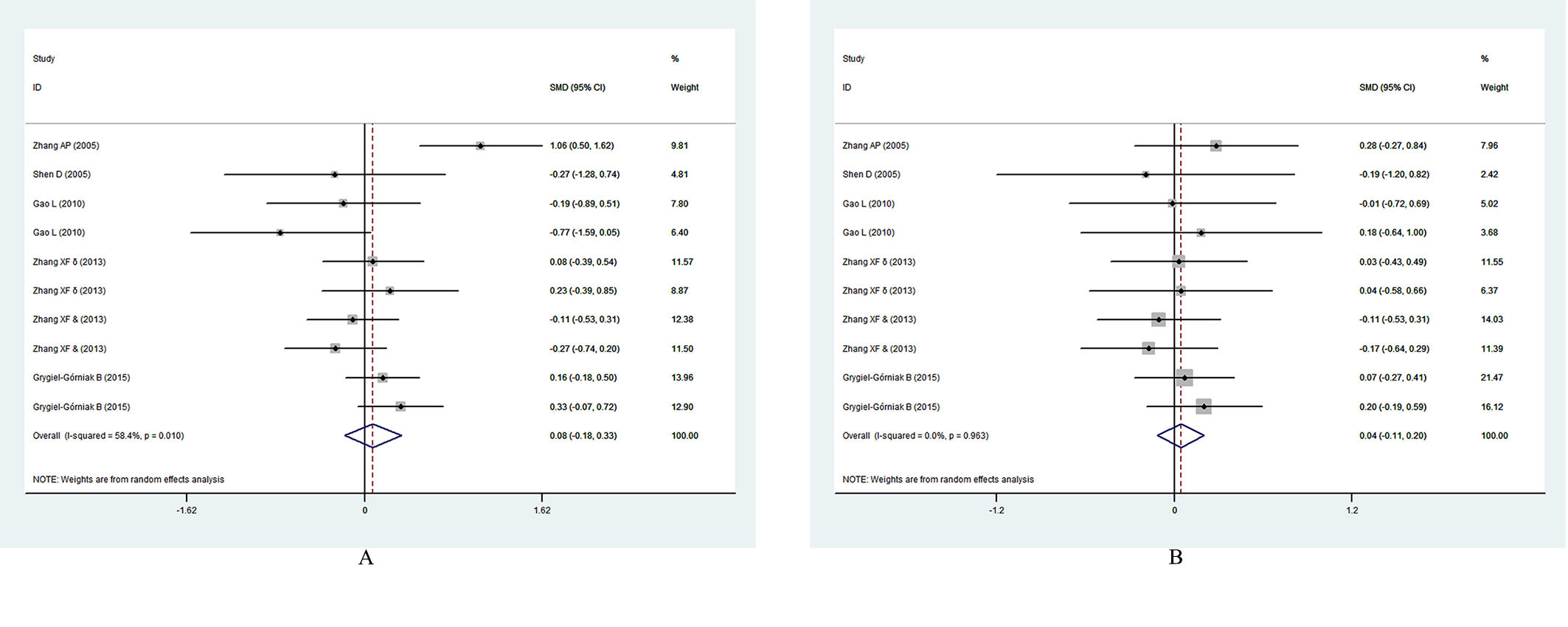

Supplement: S3 Fig — Forest plot of the association between the PPARG Pro12Ala polymorphism and the value of systolic blood pressure (A) and diastolic blood pressure (B). (TIF) [file pone.0181644.s005.tif]

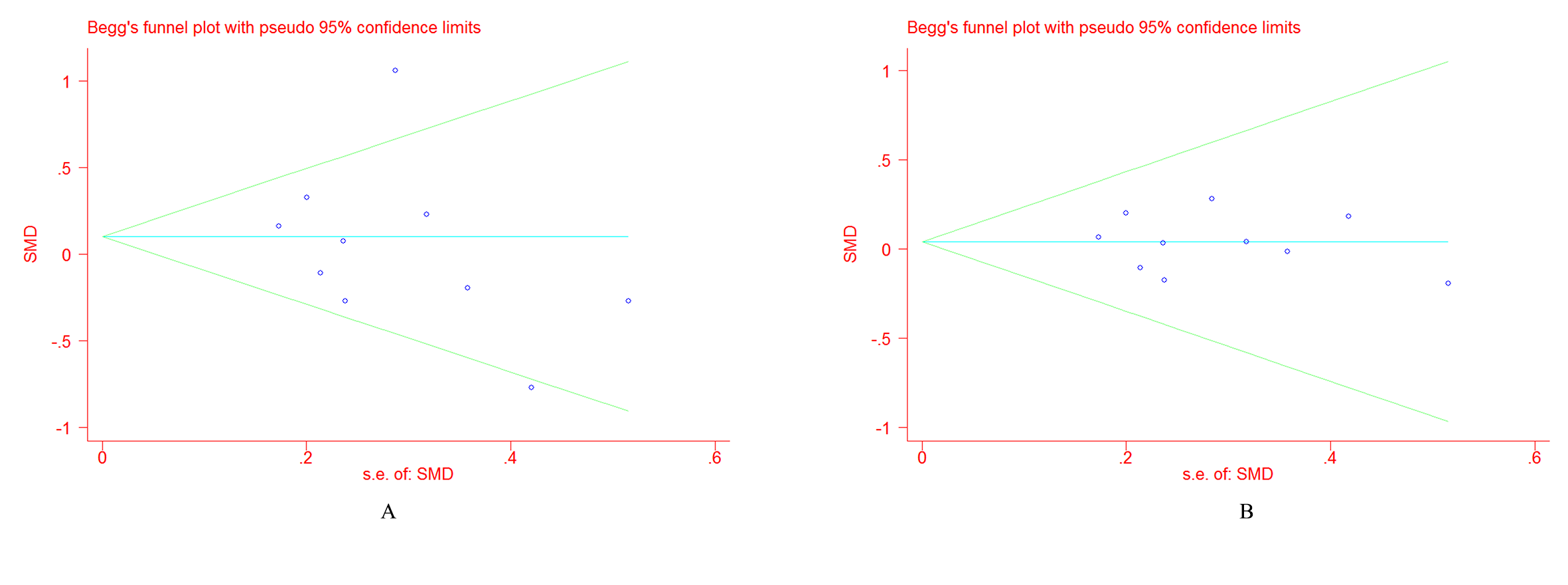

Supplement: S4 Fig — Funnel plot for the PPARG C161T polymorphism and the value of systolic blood pressure (A) and diastolic blood pressure (B). (TIF) [file pone.0181644.s006.tif]
